# Supplementary material for: Use of Angiotensin-Converting Enzyme Inhibitors and Angiotensin II Receptor Blockers During the COVID-19 Pandemic: A Modeling Analysis
Source: PLoS Comput Biol. 2020 Oct 8;16(10):e1008235. doi: 10.1371/journal.pcbi.1008235 (PMC7575117; doi:10.1371/journal.pcbi.1008235)
Supplement: Attachment — Submitted filename: reviewer_comments.docx [file pcbi.1008235.s001.docx]

**Review of “Use of Angiotensin-Converting Enzyme Inhibitors and Angiotensin Receptor Blockers During the COVID-19 Pandemic: A Modeling Analysis”**

**Summary**

Motivated by the ongoing COVID-19 pandemic, this study develops an ordinary differential equations model that represents the dynamics of the Renin Angiotensin System (RAS), binding of ACE2 with SARS-CoV-2, and the host’s acute inflammatory response. SARS-CoV-2 enters a cell via ACE2 and thus there are theoretical concerns with incomplete experimental and/or clinical evidence that the use ACE inhibitors (ACEI) and angiotensin receptor blockers (ARB) may increase the risk of infection and negative outcomes in patients who use these drugs. ACEI and ARB are frequently used to treat patients with diabetes and cardiovascular diseases such as hypertension. While the pathophysiology of COVID-19 is potentially lethal, cessation of ACEI or ARB is not ideal has it could exacerbate existing chronic health conditions in specific patient subgroups and potentially end in death. To better understand the risks/benefits of ACEI and ARB regarding COVID-19, this model presented in this study highlights these mechanisms: (1) the use of these drugs upregulates ACE2; (2) Increased expression of ACE2 increases the rate of infection; (3) soluble ACE2 circulating in the plasma may bind and inactivate SARS-CoV-2; and (4) the pro-inflammatory and anti-inflammatory actions of different isoforms of angiotensin receptor activation. Their model simulations predict that the outcomes of a patient are dependent upon the level of exposure to SARS-CoV-2, and robustness of one’s immune system.

**Major Findings**

- For individuals with mild or no chronic inflammation, ACEI may lead to worse COVID-19 outcomes (elevated viral entry outweighs the pro-inflammatory benefits of the drug)
- For the same cohort, ARBs may lead to worse COVID-19 outcomes (ARBs can disrupt the body’s inflammatory and immune response)
- For individuals with more severe inflammation, both ACEI and ARBs may improve their COVID-19 outcomes (attributed to the anti-inflammatory benefits of these drugs).

**Critiques**

- In the first section of the introduction, you say “wrecked all over the world” and believe you meant to say “wreaked havoc all over the world”
- In the second paragraph, you should fully write out “Renin Angiotensin System” before you use the acronym RAS
- In the third sentence of the second paragraph, you say “ACE2 opposes the actions of the vasoactive angiotensin II.” I was originally confused by this sentence as I incorrectly assumed that ACE2 generates Ang II via the proteolytic cleavage of Ang 1 (this is the action of ACE and not ACE2). You should give a clearer explanation of the RAS in this section.
- In the “Strength of immune response” subsection of the Results, you mention that you make the immune system more effective at reducing the viral load by doubling the parameters kpm and kpn. You should explain what these parameters represent
- In the final sentence of the “Hypertension with mild inflammation” subsection of the Results, you have a typo where you say “user” instead of “use”
- Separating the model equations and parameters into a supplemental materials document is unnecessary and makes it difficult to track down what exactly you did and understand your novel contribution. I would concatenate your supplementary materials document into the methods section.
- In Figure 1’s caption, you should explain what the arrows, solid black squares, and flat bars governing the relationships between the boxes represent.
- In the supplementary materials, it is mentioned that the ACE2 dynamics model parameters are taken from a male version of the model. Given that females are also at risk for SAR-CoV-2 infections, it would be valuable for you simulate and predict if there are sex differences in the use of ACEI and ARB with respect to COVID-19
  - If the female version of the ACE2 dynamics model is inaccurate or unusable for some reason, you should explain your reasoning for not simulating female ACE2 dynamics and be explicit in the presentation of these results by saying that they are only applicable to the male population

**Comments/Suggestions**

- It could be interesting to re-plot figure 2 so that you merge panels A1-C1, A2-C2, and A3-C3 respectively, but have the vertical axes be log-scaled. Could make for a more impactful illustration of the model dynamics.
- You could discuss what types of clinical and/or experimental data you could calibrate your model to. In a future study you may want to do a more formal model validation process (ex: parameter estimation, uncertainty quantification)
- You only model the acute immune response. Since the SARS-CoV-2 has incubation period around 2 weeks, and recovery of COVID-19 can be a long arduous process (particularly in patients with comorbidities), the chronic immune response is likely relevant. You could discuss how this might be modeled in the future
