# Supplementary material for: Use of Angiotensin-Converting Enzyme Inhibitors and Angiotensin II Receptor Blockers During the COVID-19 Pandemic: A Modeling Analysis
Source: PLoS Comput Biol. 2020 Oct 8;16(10):e1008235. doi: 10.1371/journal.pcbi.1008235 (PMC7575117; doi:10.1371/journal.pcbi.1008235)
Supplement: Attachment — Submitted filename: PLOS_review.docx [file pcbi.1008235.s002.docx]

This manuscript aims to address an urgent and important public health question – whether treatment with antihypertensive drugs ACE inhibitors or ARBs may impact the severity of COVID-19. They link a model of the RAS pathway with a model of acute inflammation, and attempt to predict recovery or death under different baseline inflammatory states, with or without ACEi or ARB treatment.

While the question is an important and timely one, there are several major concerns with this analysis. These concerns are particularly problematic given the major public health implications of suggesting changes to standard-of-care treatments for prevalent diseases like hypertension, heart disease, and kidney disease. These medications have been shown through randomized controlled trials to reduce mortality and prevent cardiovascular events. In the absence of data, modeling can be very useful to fill the gap and help identify the problem. However, one must be very careful and crtical in ones analysis, and clear in communicating its limitations.

In addition, although the authors could not have been aware of this since it was published after the manuscript was submitted, a NEJM article has shown no association of ACEi or ARB treatment with increased risk for acquiring COVID19 or for poorer outcomes. <https://www.nejm.org/doi/full/10.1056/NEJMoa2008975?query=featured_coronavirus>

My overarching concern is that the analysis uses a binary, black and white response of “recovery” or “death”, based on guessed or extremely approximate values for parameters, and overly simplified assumptions about drug effects, without any sensitivity analysis, and without any validation with actual data. I will focus on each aspect below:

1. Model structure, assumptions, and parameter:

- It is not clear that the feedback effects in which ACEi and ARBs increase renin and AngI, and subsequently increase Ang(1-7) is accounted for. At least it is not mentioned in the model equations.
- I am also confused by equation 6 – it shows a constant effect of virus on ACE2. It is not clear why this would be constant – this implies that ACE2 is cleared by SARS-CoV-2 even when no virus is present! But perhaps more concerning, the rate constant for SARS-CoV-2 binding to ACE2 (Table S1) appears to be pulled from the air.
- For some critical parameters, references are not provided. For others, a reference is provided, but it is unclear how that parameter was derived from the data in the referenced paper (e.g. ACE2 half-life from a paper that measured expression levels). No measures of uncertainty or variability in parameters are mentioned. No sensitivity analysis is conducted. Thus, it seems more likely than not that the conclusions reached are dependent on parameter choices, and that different results might be obtained with different parameter values.
- The binary model prediction (recovery/death) really hinges on the link between AT1, AT2, and Ang(1-7) on inflammatory cells andccytokines. AngII and inflammation. Thus the three parameters KAT1R, Kang17 and KAT2, are likely critically important. For instance, Table S2 lists a value for KAT1R with a reference to a paper that shows increased AT1 receptor expression following lung hypoxia. It is unclear how the parameter value was derived, but there is almost certainly huge uncertainty around it, as well as around the assumption of a linear effect on “inflammatory agents” N.
- Eq. 9 - Kburst for COVID-19 – how is it determined?
- There are many parameters in equtions 10-12 given with little justification. Are they COVID-19 specific or general to all pathogens? Either way, where did they come from?
- “The higher abundance of ACE2 would increase (i) the rate of conversion of Ang II to Ang (1-7) which is anti-inflammatory, and (ii) the rate of SARS-CoV-2 cell entry. Both rates are increased by 5 folds based on the change in ACE2 mRNA level reported in Ref. [7].” – this reference says nothing about how ACE2 affects SARS-CoV-2 entry.
- You state that “the goal of this study is to develop a mathematical model to elucidate the roles of these drugs in the pathogenesis of COVID-19.” “ Drug effects are poorly represented. You mention reduced dosage, but it is not clear what drug/dose they are modeling anyway.
- “we eliminate the effect of ACEi on ACE2 abundance.” – what assumption was made about this? No parameter is given. “ACE2 abundance” is not a parameter in the model. Are you assuming something about ACE2 production rate?

2. Model validation:

- There is a lack of data, no demonstration that the model actually produces reality in any real situation. There are several weak links in the chain from ACEi/ARB treatment to changes in viral load and inflammation.
- In the absence of virus, with the assumption regarding changes in ACE2 expression (not included in previous papers), does the model accurately reproduce change in AngI, AngII, and Ang(1-7) with ACEi or ARBs? Does it predict the different pattern of changes with different drugs?
- ACE2 dynamics – no illustration that it actually reproduces reasonable levels of plasma for intracellular ACE2, or that it appropriately represents response to pertubution. If no such data is available (it likely is not), then the sensitivity of model results to the assumed parameters must be shown.
- No validation is provided showing that the model can predict changes in inflammation in any other setting in response to an ACEi or ARB (or any other RAS perturbation).
- I recognize the challenge of model validation given limited data, but without such validation, bold clinical claims should not be made.

3. Model predictions:

- The analysis uses a binary, black and white response of “recovery” or “death”, based on guessed or extremely approximate values for parameters, and overly simplified assumptions about drug effects. “Recovery” is a very bold statement. If you are looking at this type of bistable behavior, you need to provide much more clarity about how these different states are impacted by the choice of model parameters. This sort of “yes/no” is over simplified.
- There is no way that this model can predict recovery or death. At best, it might predict some probability of resolving inflammation.
- Major concern is that the findings are way overstated, with potential to exacerbate a major public health problem right now.
- “Model simulations indicate that whether a patient should stop their ACEI or ARB treatment depends on a number of factors, including the level of existing inflammation, dosage, and
- Simulations overly simplistic – “inflammatory state simuated by increasing the generate rate of activated neutrophils” – not clear that the state was appropriate to begin with. Many ways in which inflammatory state can be altered.
