# Supplementary material for: Use of Angiotensin-Converting Enzyme Inhibitors and Angiotensin II Receptor Blockers During the COVID-19 Pandemic: A Modeling Analysis
Source: PLoS Comput Biol. 2020 Oct 8;16(10):e1008235. doi: 10.1371/journal.pcbi.1008235 (PMC7575117; doi:10.1371/journal.pcbi.1008235)
Supplement: Attachment — Submitted filename: rebuttal.docx [file pcbi.1008235.s003.docx]

**Reviewer #1**

**Critiques**

- In the first section of the introduction, you say “wrecked all over the world” and believe you meant to say “wreaked havoc all over the world”

Thank you. Corrected.

- In the second paragraph, you should fully write out “Renin Angiotensin System” before you use the acronym RAS

RAS has been defined in the first paragraph.

- In the third sentence of the second paragraph, you say “ACE2 opposes the actions of the vasoactive angiotensin II.” I was originally confused by this sentence as I incorrectly assumed that ACE2 generates Ang II via the proteolytic cleavage of Ang 1 (this is the action of ACE and not ACE2). You should give a clearer explanation of the RAS in this section.

The RAS reactions are described in the third paragraph. We added a sentence to say that “the actions of ACE2 and its homologue ACE are described below.”

- In the “Strength of immune response” subsection of the Results, you mention that you make the immune system more effective at reducing the viral load by doubling the parameters kpm and kpn. You should explain what these parameters represent

Thank you for the suggestion. We added a note to explain that kpm and kpn characterize the strength of the local immune response and the inhibitory action of the activated phagocytes.

- In the final sentence of the “Hypertension with mild inflammation” subsection of the Results, you have a typo where you say “user” instead of “use”

Thank you. Corrected.

- Separating the model equations and parameters into a supplemental materials document is unnecessary and makes it difficult to track down what exactly you did and understand your novel contribution. I would concatenate your supplementary materials document into the methods section.

We have merged the model equations and parameters into the main text. We agree that is easier to read.

- In Figure 1’s caption, you should explain what the arrows, solid black squares, and flat bars governing the relationships between the boxes represent.

Explanation added.

- In the supplementary materials, it is mentioned that the ACE2 dynamics model parameters are taken from a male version of the model. Given that females are also at risk for SAR-CoV-2 infections, it would be valuable for you simulate and predict if there are sex differences in the use of ACEI and ARB with respect to COVID-19
  - If the female version of the ACE2 dynamics model is inaccurate or unusable for some reason, you should explain your reasoning for not simulating female ACE2 dynamics and be explicit in the presentation of these results by saying that they are only applicable to the male population

We conducted simulates using parameters fitted for female as well, and model predictions are qualitatively similar. This is now stated in the Discussion.

**Comments/Suggestions**

- It could be interesting to re-plot figure 2 so that you merge panels A1-C1, A2-C2, and A3-C3 respectively, but have the vertical axes be log-scaled. Could make for a more impactful illustration of the model dynamics.

We tried the reviewer’s suggestion. But with the log scale (necessary because of the order-of-magnitude larger values in the B row), one cannot see some of the features, especially for the A and C rows. We think it’s better to stay with having the three cases separate.

- You could discuss what types of clinical and/or experimental data you could calibrate your model to. In a future study you may want to do a more formal model validation process (ex: parameter estimation, uncertainty quantification)

We added a paragraph to the Discussion to address model limitations, and how, in the future, carefully measured time-courses of viral titer and the concentrations of various immune agents would be useful in the development of a model with a more comprehensive immune response and in validating its predictions. That may be a difficult ask for a disease that does not yet have an animal model.

- You only model the acute immune response. Since the SARS-CoV-2 has incubation period around 2 weeks, and recovery of COVID-19 can be a long arduous process (particularly in patients with comorbidities), the chronic immune response is likely relevant. You could discuss how this might be modeled in the future

This is now addressed in the Discussion.

**Reviewer #2**

In addition, although the authors could not have been aware of this since it was published after the manuscript was submitted, a NEJM article has shown no association of ACEi or ARB treatment with increased risk for acquiring COVID19 or for poorer outcomes. [https://www.nejm.org/doi/full/10.1056/NEJMoa2008975?query=featured_](https://www.nejm.org/doi/full/10.1056/NEJMoa2008975?query=featured_coronavirus)coronavirus

Thank you for pointing us to this paper. Data on COVID-19 are growing fast. We have added this to the Discussion.

My overarching concern is that the analysis uses a binary, black and white response of “recovery” or “death”, based on guessed or extremely approximate values for parameters, and overly simplified assumptions about drug effects, without any sensitivity analysis, and without any validation with actual data. I will focus on each aspect below:

1. Model structure, assumptions, and parameter:

- It is not clear that the feedback effects in which ACEi and ARBs increase renin and AngI, and subsequently increase Ang(1-7) is accounted for. At least it is not mentioned in the model equations.

The model contains a simplified representation of the RAS and assumes a constant PRA. Thus, the feedback between AT1R-bound Ang II to PRA is not represented. This is now noted in the Discussion as a model limitation.

- I am also confused by equation 6 – it shows a constant effect of virus on ACE2. It is not clear why this would be constant – this implies that ACE2 is cleared by SARS-CoV-2 even when no virus is present! But perhaps more concerning, the rate constant for SARS-CoV-2 binding to ACE2 (Table S1) appears to be pulled from the air.

We apologize for the confusion regarding the binding of SARS-CoV-2 to ACE2. That term does depend on plasma SARS-CoV-2, which we left out in the original text.

We have added a sensitivity analysis to understand how variations in model parameters, including the rate constant for SARS-CoV-2 and ACE2, affect predictions.

- For some critical parameters, references are not provided. For others, a reference is provided, but it is unclear how that parameter was derived from the data in the referenced paper (e.g. ACE2 half-life from a paper that measured expression levels). No measures of uncertainty or variability in parameters are mentioned. No sensitivity analysis is conducted. Thus, it seems more likely than not that the conclusions reached are dependent on parameter choices, and that different results might be obtained with different parameter values.

We apologize for linking the wrong Wysocki et al. reference for the ACE2 half-life. It should be the 2010 paper, not 2006. As noted above, we have added a sensitivity analysis.

- The binary model prediction (recovery/death) really hinges on the link between AT1, AT2, and Ang(1-7) on inflammatory cells andccytokines. AngII and inflammation. Thus the three parameters KAT1R, Kang17 and KAT2, are likely critically important. For instance, Table S2 lists a value for KAT1R with a reference to a paper that shows increased AT1 receptor expression following lung hypoxia. It is unclear how the parameter value was derived, but there is almost certainly huge uncertainty around it, as well as around the assumption of a linear effect on “inflammatory agents” N.

We estimate these parameter values from experiments that link AT1, AT2, Ang (1-7) levels to concentrations of various inflammatory agents. We agree that there is huge uncertainty in these values. Indeed, the sensitivity analysis indicates, as the reviewer surmised, that model predictions are quite sensitive to variations in these parameters. This points to a need for experiments to be done to better quantify these relations.

- Eq. 9 - Kburst for COVID-19 – how is it determined?

This parameter is surely poorly characterized. We include this in the new sensitivity analysis. Model predictions seem rather insensitive to its variations.

- There are many parameters in equations 10-12 given with little justification. Are they COVID-19 specific or general to all pathogens? Either way, where did they come from?

Some of the parameters are general to all pathogens and were taken from Reynolds et al. (Ref. [13]). Others are COVID-19 specific, which may not have much experimental ground. We have considered those in the new sensitivity analysis.

- “The higher abundance of ACE2 would increase (i) the rate of conversion of Ang II to Ang (1-7) which is anti-inflammatory, and (ii) the rate of SARS-CoV-2 cell entry. Both rates are increased by 5 folds based on the change in ACE2 mRNA level reported in Ref. [7].” – this reference says nothing about how ACE2 affects SARS-CoV-2 entry.

The reviewer is correct that Ref. [7] reports how ACE2 mRNA level changes in response to ACE inhibition, but says nothing about how SARS-CoV-2 entry is affected. Our simulation of how ACE inhibition might affect viral entry is based on the assumption that entry rate is directly proportional to ACE2 mRNA level. That assumption is now stated explicitly.

You state that “the goal of this study is to develop a mathematical model to elucidate the roles of these drugs in the pathogenesis of COVID-19.” “ Drug effects are poorly represented. You mention reduced dosage, but it is not clear what drug/dose they are modeling anyway.

We have revised that sentence to say that “the goal of this study is to develop a mathematical model to gain insights into the effect of these drugs on the pathogenesis of COVID-19". We also added an explanation that the attenuation of the effect of ACEi or ARB (how much they affect the RAS reaction rates) can be interpreted as a lower dosage.

- “we eliminate the effect of ACEi on ACE2 abundance.” – what assumption was made about this? No parameter is given. “ACE2 abundance” is not a parameter in the model. Are you assuming something about ACE2 production rate?

We kept the rate of conversion of Ang II to Ang (1-7) and the rate of SARS-CoV-2 cell entry at baseline values. This is now stated in the text.

2. Model validation:

- There is a lack of data, no demonstration that the model actually produces reality in any real situation. There are several weak links in the chain from ACEi/ARB treatment to changes in viral load and inflammation

We agree with the reviewer that the lack of data is an issue for this novel disease that does not yet have an animal model. Below we have sought to address issues raised by the reviewer on ACEi/ARB simulations.

- In the absence of virus, with the assumption regarding changes in ACE2 expression (not included in previous papers), does the model accurately reproduce change in AngI, AngII, and Ang(1-7) with ACEi or ARBs? Does it predict the different pattern of changes with different drugs?

The RAS model with the virus was taken from Ref. [10]. There we simulated ACEi and ARB, and model responses were validated.

- ACE2 dynamics – no illustration that it actually reproduces reasonable levels of plasma for intracellular ACE2, or that it appropriately represents response to pertubution. If no such data is available (it likely is not), then the sensitivity of model results to the assumed parameters must be shown.

Parameters characterizing ACE2 dynamics are now considered in a sensitivity study.

- No validation is provided showing that the model can predict changes in inflammation in any other setting in response to an ACEi or ARB (or any other RAS perturbation).

The RAS model on which the present model is based has been applied to ACEi and ARBl (Leete et al. 2018). Those simulations have been validated. The acute inflammatory response model has also been separately validate (Reynolds et al. 2006) although that was done independently of a RAS.

- I recognize the challenge of model validation given limited data, but without such validation, bold clinical claims should not be made.

We completely agree with the reviewer, and have revised the manuscript to eliminate any bold clinical claims.

3. Model predictions:

- The analysis uses a binary, black and white response of “recovery” or “death”, based on guessed or extremely approximate values for parameters, and overly simplified assumptions about drug effects. “Recovery” is a very bold statement. If you are looking at this type of bistable behavior, you need to provide much more clarity about how these different states are impacted by the choice of model parameters. This sort of “yes/no” is over simplified.
- There is no way that this model can predict recovery or death. At best, it might predict some probability of resolving inflammation.

We agree with the reviewer that the model cannot predict recovery or death. We have rephrased the interpretation of model prediction to “tissue recovery” (which is not the same as “recovery” of a patient) and “sustained tissue damage.”

- Major concern is that the findings are way overstated, with potential to exacerbate a major public health problem right now.

Agreed. We don’t want that. We hope the revised manuscript is much improved in this regard.

- “Model simulations indicate that whether a patient should stop their ACEI or ARB treatment depends on a number of factors, including the level of existing inflammation, dosage, and
- Simulations overly simplistic – “inflammatory state simuated by increasing the generate rate of activated neutrophils” – not clear that the state was appropriate to begin with. Many ways in which inflammatory state can be altered.

We agree that our approach to simulate an inflammatory state is simplistic. We now acknowledge that, as the reviewer noted, “inflammatory status can be altered by a number of factors” and that we simulate an inflammatory state by increasing the generate rate of activated neutrophils for simplicity.
