# Supplementary material for: Use of Angiotensin-Converting Enzyme Inhibitors and Angiotensin II Receptor Blockers During the COVID-19 Pandemic: A Modeling Analysis
Source: PLoS Comput Biol. 2020 Oct 8;16(10):e1008235. doi: 10.1371/journal.pcbi.1008235 (PMC7575117; doi:10.1371/journal.pcbi.1008235)
Supplement: Attachment — Submitted filename: reviewer_comments_resub.docx [file pcbi.1008235.s004.docx]

**Review of “Use of Angiotensin-Converting Enzyme Inhibitors and Angiotensin Receptor Blockers During the COVID-19 Pandemic: A Modeling Analysis” Resubmission**

Overall, the authors have done a great job responding to reviewer comments. I think this is a timely and interesting study, and I look forward to seeing it published. I have one major critique regarding the clarity of how “tissue recovery” is presented.

**Critiques**

- In the first section of the introduction, you misspelled “wreaked” as “wrecked”
- Virtually all your results are based on if tissue “recovers” from damage or not, however you do not provide a clear definition of “recovery.” I have surmised “recovery” refers to the tissue damage variable (D) converging toward zero. This should be stated early in Results section.
  - I would suggest adding a few sentences or a short paragraph at the beginning of the results section to outline how you intend to use the behavior of D and other key model variables to assess the impact of COVID-19 and ACEi/ARB.
  - I suspect that there may be a parameter bifurcation, or basin of initial conditions that determines the steady state value (critical point) of D. I would not recommend you investigate this right now (given the urgency of the COVID-19 situation), but exploring this could be interesting in a follow up study
- Throughout the paper you refer to equations in the supplemental materials, but you have merged the supplemental methods into the primary body of the paper. This is likely an oversight of that merging process, but should be corrected to avoid confusion

**Suggestions**

- Consider moving the Methods section to before the Results section. You actively refer to your model (parameters, equations, variables, etc.) in the Results and it can be difficult to follow what you are referring to before having read the Methods section
- Here is a recent paper investigating the role of neutrophil extracellular traps in the context of COVID-19:

<https://insight.jci.org/articles/view/138999>

Simply put, elevated extracellular traps from neutrophils have been observed in COVID-19 and correlates with various biomarkers and worse patient outcomes. I do not think it is essential for you to reference this study, but it could be worth referencing in the Introduction and/or Discussion
