# Supplementary material for: Use of Angiotensin-Converting Enzyme Inhibitors and Angiotensin II Receptor Blockers During the COVID-19 Pandemic: A Modeling Analysis
Source: PLoS Comput Biol. 2020 Oct 8;16(10):e1008235. doi: 10.1371/journal.pcbi.1008235 (PMC7575117; doi:10.1371/journal.pcbi.1008235)
Supplement: Attachment — Submitted filename: rebuttal2.docx [file pcbi.1008235.s005.docx]

**Reviewer #1**

**Critiques**

- In the first section of the introduction, you misspelled “wreaked” as “wrecked”

Thank you. Corrected.

- Virtually all your results are based on if tissue “recovers” from damage or not, however you do not provide a clear definition of “recovery.” I have surmised “recovery” refers to the tissue damage variable (D) converging toward zero. This should be stated early in Results section.

The definition of “recovery” was given at the end of Methods, which is probably too late. We have moved that definition to the Results section.

- I would suggest adding a few sentences or a short paragraph at the beginning of the results section to outline how you intend to use the behavior of D and other key model variables to assess the impact of COVID-19 and ACEi/ARB.

Done

- I suspect that there may be a parameter bifurcation, or basin of initial conditions that determines the steady state value (critical point) of D. I would not recommend you investigate this right now (given the urgency of the COVID-19 situation), but exploring this could be interesting in a follow up study.

Yes we pursue this direction in a follow-up study.

- Throughout the paper you refer to equations in the supplemental materials, but you have merged the supplemental methods into the primary body of the paper. This is likely an oversight of that merging process, but should be corrected to avoid confusion

Thank you. Corrected.

**Suggestions**

- Consider moving the Methods section to before the Results section. You actively refer to your model (parameters, equations, variables, etc.) in the Results and it can be difficult to follow what you are referring to before having read the Methods section

We agree, however this is the section ordering in PLoS Comput. Biol.

- Here is a recent paper investigating the role of neutrophil extracellular traps in the context of COVID-19: <https://insight.jci.org/articles/view/138999>

Simply put, elevated extracellular traps from neutrophils have been observed in COVID-19 and correlates with various biomarkers and worse patient outcomes. I do not think it is essential for you to reference this study, but it could be worth referencing in the Introduction and/or Discussion

Thank you for the suggestion. This is an interesting study. We have cited it in the Discussion.

**Reviewer #2**

1. There are a lot of symbols and abbreviations in this manuscript. A list of all symbols and their definitions would really help the reader.

Symbols and corresponding definitions have been added to Tables 3 and 4.

2. Even if you make a list, it would be helpful to state in the Table caption (or header row) of Table 2 that P and D refer to peak viral load and tissue damage.

Done.

3. What do the dashed lines mean? No change? Why not just put 0%?

Changes in peak viral load and tissue damage are reported only for cases that achieve tissue recovery. When recovery isn’t possible, viral load and damage are large.

4. I don’t fully understand the table. For instance, for S_ACE2m, dividing it by 2 increases tissue damage 133%, but also leads to recovery? This needs to be explained a little more.

The baseline parameters yield recovery. Reducing $\boldsymbol{S}_{\text{ACE2m}}$ by half more than doubles tissue damage and makes it harder to recovery. Nonetheless, unlike some other cases, the model manages to recover, just barely.

5. You say that “in particular, this analysis indicates that model results are particularly sensitive to the pro-inflammatory effect of AT1R-bound Ang II (AT1R) and to the anti-inflammatory effect of ACE2 (ACE2p).” I can’t tell this from the table. Their values for delta P and delta D have dashes, so it is not clear what the sensitivity is.

Model results are particularly sensitive to changes in these parameters, because in each case 10% variation in either direction results in sustained tissue damage (thus dashes for delta P and delta D). We now note that delta P and delta D are reported only for cases with tissue recovery. Hopefully that eliminates the confusion here.

6. You continue that “Interestingly, a sufficiently large deviation in these parameters from their baseline values in either direction would result in sustained tissue damage.” This seems really unintuitive and difficult to understand. Why would increasing the anti-inflammaotry effect of ACE2 (+10%) make recovery LESS likely? Why would decreasing the inflammatory effect of KAT1R make recovery less likely? These findings need to be explained further.

This is indeed an interesting result that highlights the role of inflammation as a double-edged sword. An attenuation in the inflammatory response simultaneously attenuates tissue damage and impedes the system’s defense against pathogens. With the invasion of a new virus, attenuation of the inflammatory defense may yield sustained tissue damage.

7. Some key parameters are missing from this table – Especilaly Kdn and ud – the rate constants in equation 12 for tissue damage. These relative rates are likely to be hugely important in determining how D changes over time, and thus whether recovery occurs. They should be added.

These parameters have been added to the table.
